# Breast reconstruction after complications following breast augmentation with massive filler injections

**DOI:** 10.1097/MD.0000000000021516

**Published:** 2020-08-14

**Authors:** Hyeon Jo Kim, Seong Joo Lee, Ju Ho Lee, Se Ho Shin, Seong Hwan Kim, Jae Hyun Kim, In Suck Suh

**Affiliations:** Department of Plastic and Reconstructive Surgery, Kangnam Sacred Heart Hospital, Hallym University College of Medicine, Seoul, Republic of Korea.

**Keywords:** breast reconstruction, complication, filler, mammoplasty, polyacrylamide gel

## Abstract

**Introduction:**

Breast filler injections are less commonly used due to their associated complications, such as pain and foreign body reactions. Yet, these fillers are often administered illegally, resulting in aesthetic or life-threatening complications. These are treated by removing the foreign material, and the breasts are reconstructed using silicone implants or autologous tissue/fat injection.

**Patient concerns:**

Case 1. A 45-year-old woman with polyacrylamide gel injections in both breasts visited our clinic for breast pain and tenderness. Grade I ptosis was observed in each breast, without skin necrosis and discoloration. Case 2. A 51-year-old woman, with unknown breast filler injections, visited our clinic for painful masses. Intraoperatively, massive amounts of foreign material had severely infiltrated the nearby tissues; thus, an immediate breast reconstruction could not be performed. Three months later, severe deformities including shrinkage and irregular breast skin surfaces were observed.

**Diagnosis:**

Case 1. Multiple cystic lesions, fluid collection in the retromammary spaces, and diffuse infiltration were observed on mammography, computed tomography, and ultrasonography. Case 2. Multiple cystic lesions, calcified areas, and diffuse infiltrations in the axillae and retromammary spaces were observed on mammography, computed tomography, and ultrasonography.

**Interventions:**

Case 1. The foreign material was removed and the breasts were reconstructed using silicone implants into subpectoral pocket with acellular dermal matrices (Alloderm, Lipocell Corporation). Case 2. A delayed reconstruction was undertaken using silicone implants covered by latissimus dorsi muscle flaps, 3 months after the foreign material removal.

**Outcomes:**

Case 1. The foreign material was removed and there were no complications such as foreign body reaction, capsular contracture. Ptosis was corrected and both breasts were symmetric with proper projection. Case 2. Residual foreign material was removed and there were no complications such capsular contracture, implant malposition.

**Conclusion:**

Massive injections of foreign materials into the breast can cause severe infiltration and associated foreign body reactions. By a near-complete removal of the foreign materials and breast reconstruction using silicone implants, we achieved satisfactory results, without complications such as wound disruption, capsular contracture, and implant malposition.

## 1 Introduction

Breast augmentation is one of the most popular aesthetic surgeries in the world. There are 2 methods of breast augmentation; one involves the insertion of implants (such as silicone), while the other involves the injection of fillers, such as autologous fat and biomaterials. Breast augmentation by implant insertion requires general anesthesia and is highly dependent upon the surgeon's experience. Furthermore, repetitive surgeries are required for revising the final results. Therefore, surgeons have explored simpler procedures using filler injections; these include the use of autologous fat and biomaterials. Autologous fat injection has no foreign body reactions; however, problems such as fat absorption, necrosis, and calcification exist.^[1,2]^ Moreover, it requires a donor site, thereby having a limited scope in thin patients. Among biomaterials, paraffin, petrolatum, vegetable oil, lanolin, bee's wax, silicone gel, and polyacrylamide gel (PAAG) have been used. These materials are problematic, because they can be obtained and used easily. Most of these are banned for use as breast fillers in western countries, due to the complications arising from foreign body reactions, such as inflammation, fibrosis, granuloma, and skin necrosis.^[3–5]^ Yet, breast augmentation using such biomaterial fillers is still performed privately and in illegal ways, and many patients have suffered from complications after it. In this paper, the authors report 2 cases of patients who received filler injections (PAAG and a filler of unknown component) for breast augmentation and later developed complications in the breasts.

## 2 Patients and methods

This is a retrospective case study of 2 patients who visited our clinic (the Department of Plastic and Reconstructive Surgery) from February 2018 to February 2019. One patient had an unknown material injected into both breasts, while the other had PAAG injected into both breasts. Ultrasonography, mammography, computed tomography (CT), and laboratory blood tests were performed. Immediate and delayed breast reconstruction were separately undertaken in the patients, followed by a near-complete removal of the foreign materials (fillers). All treatments were performed by the same plastic surgeon. The patients were of Korean or Chinese descent. The study protocol was approved by the Institutional Review Board (number: 2019-10-022). The patients have provided informed consent for publication of the case. All procedures in the study were performed in accordance with the ethical standards of the institutional and/or national research committee and with the 1964 Declaration of Helsinki and its later amendments or comparable ethical standards.

## 3 Case reports

### 3.1 Case 1, immediate breast reconstruction

**Figure 1:**
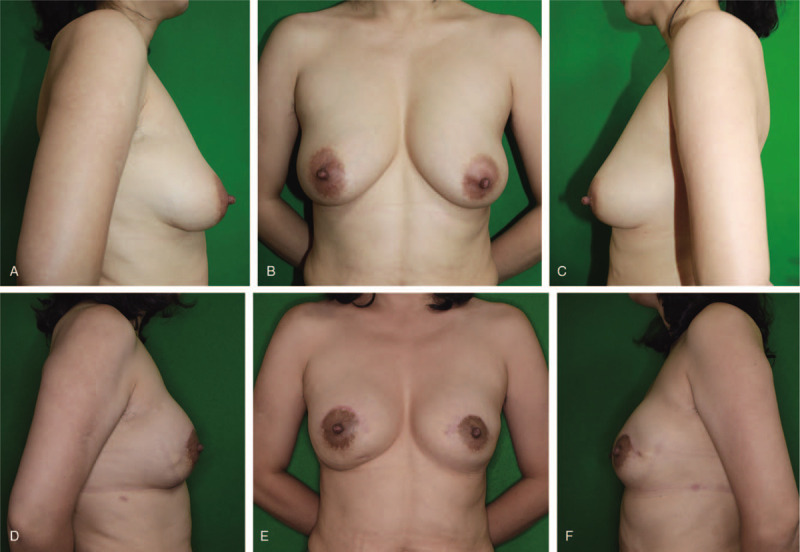
(A, B, C) Preoperation, the height of the nipples is different. Asymmetry and Grade I ptosis were observed for both breasts, without a skin color change. (D, E, F) Six months after the operation, symmetry of both breasts was achieved and the ptosis was corrected with minimum scarring. No hematoma, seroma, wound infection, and capsular contracture were observed.

**Figure 2:**
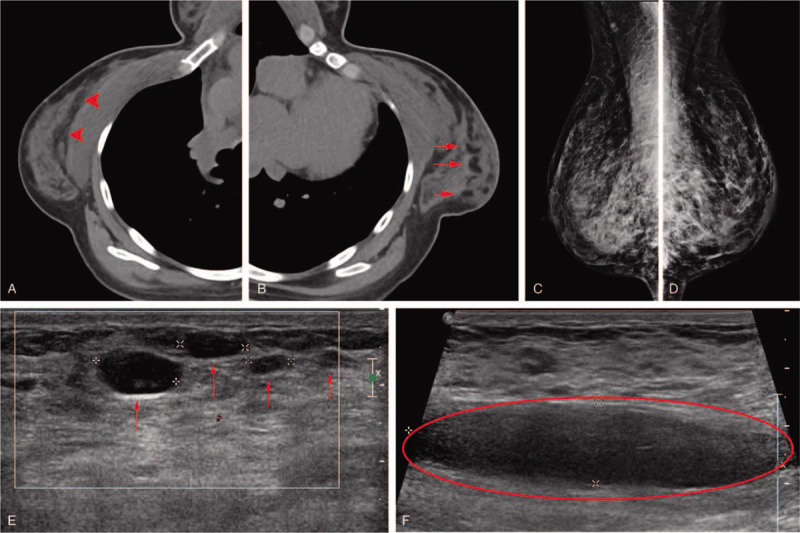
(A, B) Computed tomography scan. Multiple cystic lesions and fluid-filled areas were observed in both breasts (arrow). (C, D) Mammography. (E) Ultrasonography. Multiple hypoechoic lesions suggestive of cystic masses were observed in the breast parenchyma (arrow). (F) Ultrasonography. Fluid collection was observed in the retromammary space (circle).

**Figure 3:**
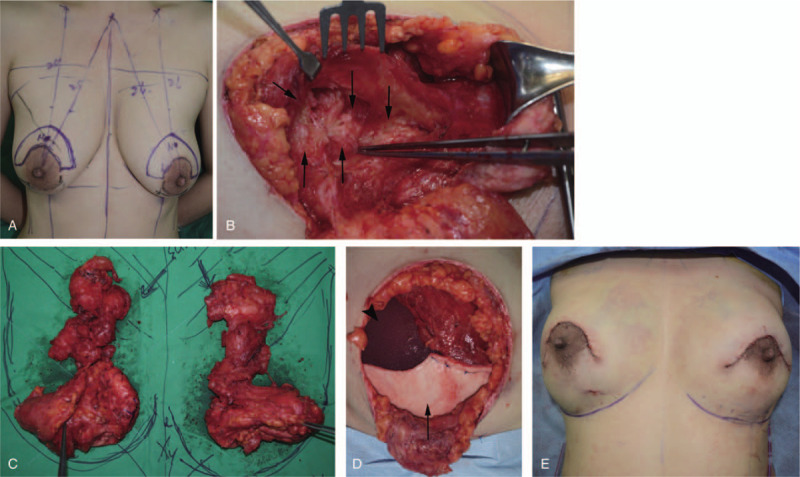
(A) Preoperative design. A horse shoe-shaped incision was made right above the nipple areolar complex for mastopexy. (B) PAAG infiltration in the pectoralis muscles (arrow). (C) All the breast parenchyma and masses infiltrated with PAAG were eradicated (305 g). (D) Round-shaped silicone implants (250 cc; Memory gel, Mentor) were inserted beneath the pectoralis major muscle (arrow head). The residual silicone implants revealed by muscle excision were covered with the acellular dermis matrix (Alloderm, Lipocell corporation) (arrow). (E) Immediately after the operation. PAAG = polyacrylamide gel.

A 45-year-old woman visited our clinic for multiple painful masses in both breasts lasting for 4 months. The patient had received PAAG injections into each breast, 13 years ago, in China. Upon palpation, multiple firm masses were identified in both breasts, accompanied by pain and tenderness. Grade I ptosis was observed in each breast, without skin necrosis and discoloration (Fig. 1A–C). C-reactive protein levels and erythrocyte sedimentation rate were within the normal range. Multiple cystic lesions, fluid collection in the retromammary spaces, and diffuse infiltration were observed on mammography, CT, and ultrasonography (Fig. 2). Due to complications arising from filler injections, we recommended breast reconstruction by autologous tissue transfer; however, the patient was worried about scarring at the donor site and feared a long operation time. Therefore, a foreign body removal surgery was performed and the breasts were reconstructed using silicone implants. Additionally, for ptosis correction, mastopexy was performed by making a horseshoe-shaped incision right above the nipple-areolar-complexes (NACs) (Fig. 3A). The PAAG filler had diffusely infiltrated into the parenchyma, subpectoral spaces, and partially into the muscles (Fig. 3B). While it was impossible to remove all the infiltrated tissues completely, a near-complete removal of the foreign material was achieved. Thereafter, 250 cc round-shaped silicone implants (Memory gel, Mentor) were inserted under the pectoralis major muscles. The exposed implants (which were not covered by the muscles) were covered with acellular dermal matrices (Alloderm, Lipocell corporation) (Fig. 3D). Six months after the operation, no complications, such as hematoma, seroma, capsular contracture, and foreign body reactions were observed. Minimal scarring was visualized. Ptosis was corrected and the aesthetic results included symmetrical breasts with proper projection (Fig. 1D–F).

### 3.2 Case 2, delayed breast reconstruction

**Figure 4:**
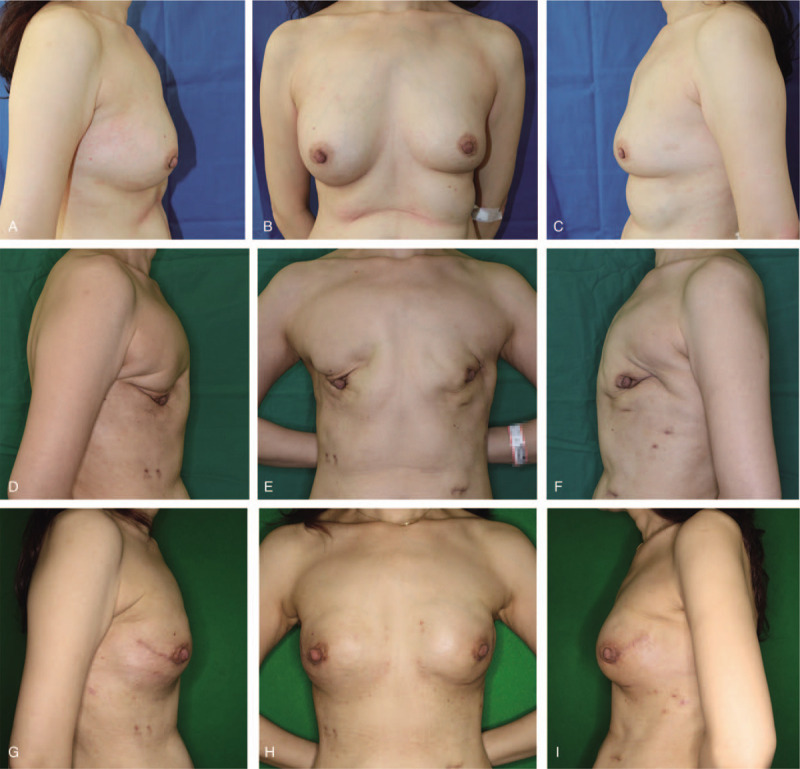
(A, B, C) Before operation. Asymmetry of both breasts and filler migration to the axillae were observed. (D, E, F) Three months after subcutaneous mastectomy, both breasts were distorted and shrunk, with an intact nipple areolar complex. (G, H, I) One month after breast reconstruction with latissimus dorsi muscle flap and silicone implants. Symmetry of both breasts and inframammary folds were achieved with minimum scarring.

**Figure 5:**
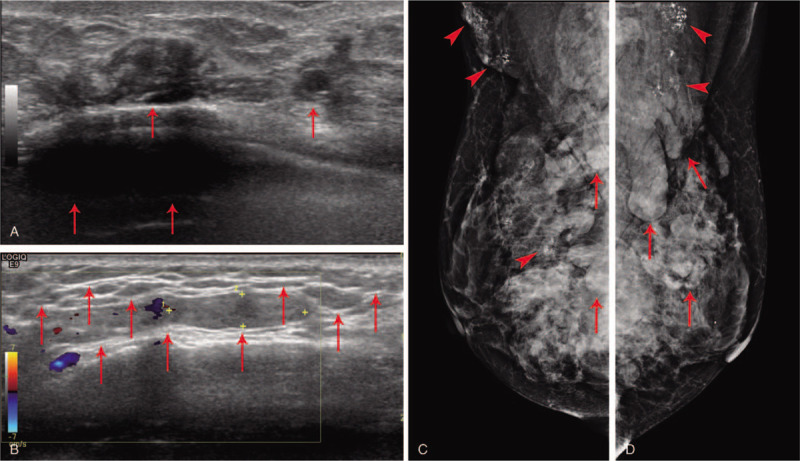
(A, B) Ultrasonography. Multiple hypoechoic lesions suggestive of cystic masses and diffuse infiltrations were observed (arrow). (C, D) Mammography. Multiple, irregularly-shaped masses (arrow) and calcification (arrow head) were observed in both breasts and axillas.

**Figure 6:**
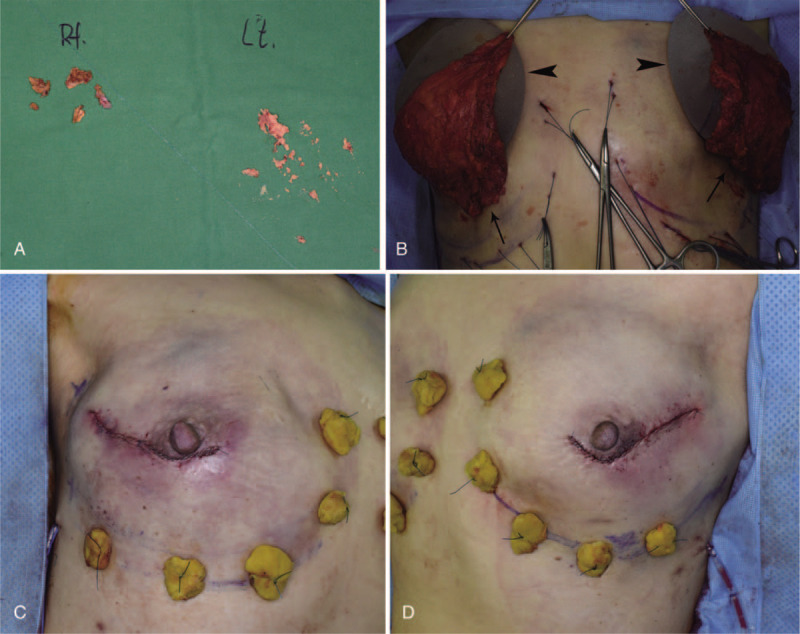
(A) Residual foreign materials, such as plaster powder, were removed intraoperatively from both axillas. (B) Anatomically-shaped silicone implants (255 cc; Memory gel, Mentor) (arrow head) were covered by latissimus dorsi muscle flaps (arrow). (C, D) Immediately after operation.

A 51-year-old woman visited our clinic with painful masses and asymmetry in both breasts. She had received injections of unknown material into each breast, 20 years ago, in China. Palpation revealed multiple masses in both breasts and the axillae, with pain and tenderness. The breasts were asymmetrical and no skin discoloration or necrosis was observed (Fig. 4A–C). The C-reactive protein levels and the erythrocyte sedimentation rate were within the normal range. Multiple cystic lesions, calcified areas, and diffuse infiltrations in the axillae and retromammary spaces were observed on mammography, CT, and ultrasonography (Fig. 5). A NAC sparing subcutaneous mastectomy (general surgery), followed by breast reconstruction with silicone implants (plastic and reconstructive surgery) were planned. However, during the NAC sparing subcutaneous mastectomy, we observed that a large amount of foreign material, such as plaster powder, had infiltrated the breast parenchyma and subpectoral spaces (Fig. 6A). Therefore, breast reconstruction could not be performed and we changed our plan from immediate reconstruction to delayed reconstruction. Three months later, the patient revisited our clinic for breast reconstruction; severe deformities including shrinkage and irregular breast skin surfaces were observed (Fig. 4D–F). Because of severe foreign material infiltration, we recommended breast reconstruction by autologous tissue transfer; however, the patient was worried about donor site scarring and feared a long operation time. Moreover, the patient was not eligible for a transverse rectus abdominis musculocutaneous flap, because the patient was thin and did not have enough soft tissue on abdomen. Therefore, we performed a breast reconstruction using silicone implants and latissimus dorsi muscle flaps. During the operation, we observed residual foreign materials in both axillae. The pectoralis major and minor muscles were severely atrophied. After removing as much of foreign material as possible, 255 cc anatomical-shaped silicone implants (Memory gel, Mentor) were inserted and covered by the latissimus dorsi muscle flaps (Fig. 6B). The postoperative course was uneventful. Two months after operation, there were no complications such as foreign body reaction, capsular contracture, and implant malposition (Fig. 4G–I).

## 4 Discussion

For breast augmentation, saline or silicone implants are commonly inserted into the subglandular or subpectoral spaces. This procedure is the safest and most preferred; however, patients generally desire a simpler procedure, due to fear of surgery, complications, and a relatively longer postoperative recovery. Thus, filler injections for breast augmentation have been developed as a simpler alternative; these are widely used in Russia, Eastern Europe, and China. Various kinds of fillers are used, including autologous fat, paraffin, petrolatum, silicone gel, and PAAG. Filler injections can be administered under local anesthesia, and the procedure is simpler than the insertion of implants such as silicone and saline bags. In some cases, filler injections can be administered for revisional purposes in patients who underwent breast augmentation with implants.^[6]^ Among the filler materials, PAAG has been widely used for the past decades in Russia, Eastern Europe, and China. It is a colorless, water-soluble, sticky material that is composed of acrylamide and methacrylamide.^[7]^ However, its use for the purpose of soft tissue augmentation was banned, because of complications such as pain, masses, migration, asymmetry, and infection. PAAG can easily translocate to the subcutaneous tissues. It can absorb body fluids and exudates, forming nutrient-rich substrates that can serve as a good medium for bacterial growth. In some cases, systemic infection leading to life-threatening septic shock can occur.^[8]^ Once the infection sets, treatment is very difficult and recurrence is frequently observed.^[4,9]^ Furthermore, gravity and muscle contraction can lead to the migration of PAAG from the injection area to the chest wall, abdominal wall, back, and even the perineum and thighs.^[10]^ It is very difficult to detect breast cancer in the early stages, when it is covered by the induration of the injected PAAG and inflammation; PAAG injection can affect the outcomes of breast cancer diagnosis and prognosis.^[11]^ In addition to PAAG, other filler materials such as paraffin and silicone gels also cause serious complications, such as foreign body granuloma and skin necrosis. Paraffin, first discovered in 1830, is a purified mineral oil whose primary purpose is to serve as a vehicle for oil-soluble substances.^[12]^ Liquid paraffin injections can cause an adverse condition called paraffinoma, which is a chronic, granulomatous, inflammatory reaction.^[13]^ It is clinical significant, because it can infiltrate into nearby structures and initiate several clinical symptoms, such as pain, palpable masses, and skin ulcerations that can lead to skin defects.^[13–15]^ Typical features of paraffinoma in hematoxylin and eosin staining include round or oval shaped, empty, pseudo-cysts encompassed by lymphocytes, epithelioid cells, and giant cells.

Radiological imaging provides an important basis for the diagnosis and treatment of complications after filler injections.^[10]^ Imaging modalities include magnetic resonance imaging (MRI), color Doppler ultrasound, and mammograms. MRI has the highest sensitivity among these methods and can be used to form an accurate image of the filler distribution. MRI often shows foreign body shadows of different sizes, irregular shapes, uneven density, and vaguely defined boundaries. Color Doppler ultrasonography is rapid and simple, and can be used intraoperatively to guide the removal of the injected filler. The filler is usually scattered in different layers, such as the breast parenchyma, subcutaneous tissue, retromammary space, and muscle. Thus, for the treatment of complications after a filler injection, the foreign material, infected tissue, and the necrotic tissue must be removed, taking care to preserve as much of the healthy tissue as possible. A pathological examination should then be conducted. In aesthetic breast reconstruction, preservation of the NAC is very important, because it provides a superior aesthetic result as compared to NAC reconstruction after excision. Nipple skin perfusion is predominantly from the skin. Only one-thirds of the vessels to the nipple travel within the duct bundle, whereas two-thirds travel within the nipple skin^[16]^; areolar perfusion is almost exclusively through the skin vessels. Thus, it is important to preserve the skin flap around the NAC. Excessive retraction on the areolar skin can increase the rates of skin necrosis, change the areolar contour, and cause NAC deviations; therefore, a gentle and atraumatic retraction is necessary.^[17,18]^ After removal of the foreign material, breast reconstruction can be performed using silicone implants, autologous fat injections, or autologous tissue transfer. Because fat injection following filler removal carries a high infection risk, it should ideally be conducted after 3 to 6 months of follow-up. The advantage of autologous fat transplantation is its ability to repair a variety of breast shape deformities; however, it may need to be repeated several times. The plane under the pectoralis major is preferred for the placement of the prosthesis; this avoids the prosthesis's contact with the residual foreign material, thereby reducing the chances of infection. Some patients suffer from serious damage to the pectoralis major and soft tissues attached to the sternum. For such patients, decellularized allogeneic dermis can be used to repair the local tissue, remodel the location of the submammary fold, and provide a more stable support for the prosthesis.^[10]^

In this paper, we recommended breast reconstruction by autologous tissue transfer in 2 patients, because the foreign material infiltration and associated inflammation was severe. Autologous tissue transfer is ideal for the treatment of complications arising from foreign material injection; however, because a majority of the patients reject this procedure for fear of donor site scaring and long operation times, breast reconstruction using silicone implants is commonly performed. In Case 1, while some infiltration was observed in the pectoralis muscles, they were preserved as much as possible during the operation. Thereafter, silicone implants could be placed under them and the exposed residual implants could be covered with acellular dermal matrix. In Case 2, the patient was not eligible for a transverse rectus abdominis musculocutaneous flap, because the patient was thin and did not have enough soft tissue on abdomen. Moreover, a pocket sufficient for implant insertion was absent due to severe pectoralis muscle atrophy. Thus, we covered the silicone implants using a latissimus dorsi muscle flap. The breast skin was distorted, with no necrosis, and expanded enough to create a natural breast contour after the surgery. In both patients, the outcome was satisfactory, without any complications such as wound infection, capsular contracture, and malposition.

## 5 Conclusion

Massive breast filler injections can cause severe infiltration, foreign body reactions, and associated symptoms such as palpable masses, pain, tenderness, and breast asymmetry. Complete removal of the injected foreign materials, while ideal, is impossible to achieve due to diffuse infiltration. Therefore, by a near-complete removal of the foreign materials, followed by an immediate and/or delayed breast reconstruction using silicone implants, we can achieve satisfactory results without any complications such as wound disruption, capsular contracture, and implant malposition.

## Author contributions

XXXX.
